# LRP4 and Agrin Are Modulated by Cartilage Degeneration and Involved in β-Catenin Signaling in Human Articular Chondrocytes

**DOI:** 10.3390/ijms26031007

**Published:** 2025-01-24

**Authors:** Shuichi Naniwa, Keiichiro Nishida, Aki Yoshida, Yoshihisa Nasu, Ryuichi Nakahara, Takashi Ohtsuki, Yoshifumi Hotta, Noriyuki Shimizu, Chinatsu Ichikawa, Deting Lin, Noriaki Otsuka, Toshifumi Ozaki

**Affiliations:** 1 Department of Orthopaedic Surgery, Section of Medicine, Division of Medicine, Dentistry and Pharmaceutical Sciences, Graduate School of Medicine, Okayama University, 2-5-1, Shikata-cho, Kita-ku, Okayama 700-8558, Japan; me19061@s.okayama-u.ac.jp (S.N.); ayo@md.okayama-u.ac.jp (A.Y.); me422049@s.okayama-u.ac.jp (N.S.); cntickw@icloud.com (C.I.); ldt773932009@gmail.com (D.L.); pbkn1mr4@s.okayama-u.ac.jp (N.O.); 2 Locomotive Pain Center, Okayama University Hospital, 2-5-1, Shikata-cho, Kita-ku, Okayama 700-8558, Japan; nasu_y@flute.ocn.ne.jp (Y.N.); pikumin55@gmail.com (R.N.); 3 Department of Medical Technology, Graduate School of Health Sciences, Okayama University, 2-5-1, Shikata-cho, Kita-ku, Okayama 700-8558, Japan; sohmaohtsuki@yahoo.co.jp; 4 Department of Orthopaedic Surgery, Sayo Central Hospital, 3529-3, Sayo-cho 679-5383, Japan; yoshifumi19870707@icloud.com; 5 Department of Orthopaedic Surgery, Faculty of Medicine, Dentistry and Pharmaceutical Sciences, Okayama University, 2-5-1, Shikata-cho, Kita-ku, Okayama 700-8558, Japan; tozaki@md.okayama-u.ac.jp

**Keywords:** osteoarthritis, chondrocyte, mechanical stress, LRP4, Agrin, β-catenin, SOX9

## Abstract

We investigated the roles of low-density lipoprotein receptor-related protein (LRP) 4 and its ligand Agrin in the pathophysiology of cartilage degeneration. Immunohistochemical analysis of human normal articular cartilage and cartilage tissues from patients with osteoarthritis (OA) obtained during surgery of the knee joint showed marked LRP4 expression in the early stages of OA, which then decreased with cartilage degeneration, whereas Agrin was consistently increased with cartilage degeneration. In normal human articular chondrocytes (NHACs), mild cyclic tensile strain (CTS) (0.5 Hz, 5% elongation, 2 h) increased the expression of LRP4 and aggrecan (ACAN), while intense CTS (0.5 Hz, 10% elongation, 6 h) increased the expression of Agrin without affecting LRP4 expression. Treatment with recombinant human (rh) Agrin downregulated the mRNA expression of LRP4 and ACAN, but upregulated the expression of LRP5/6, SRY-box transcription factor 9 (SOX9), Runt-related transcription factor 2 (RUNX2), and a disintegrin and metalloproteinase with thrombospondin motifs-4 (ADAMTS-4). Immunocytochemistry and Western blot analysis showed that rhAgrin treatment upregulated the expression of β-catenin and SOX9. Agrin knockdown by si*AGRN* transfection partially reduced the nuclear protein expression of β-catenin, which was increased with intense CTS. LRP4 knockdown by si*LRP4* transfection increased the expression of LRP5/6, SOX9, RUNX2, ADAMTS-4, and Agrin. These results suggested that intense CTS increases the expression of Agrin, which might interfere with the role of LRP4 in the inhibition of LRP5/6 and their downstream β-catenin signaling, leading to cartilage degeneration.

## 1. Introduction

Osteoarthritis (OA) is the most common joint disease characterized by the degradation of articular cartilage, remodeling of subchondral bone, osteophyte formation, synovial inflammation, degeneration of ligaments and menisci, and hypertrophy of the joint capsule [1,2]. The number of patients affected is continuing to rise and will lead to a substantial increase in the future [3]. Since OA causes disability in adult individuals due to chronic pain and loss of joint mobility, as well as a significant medical economic burden [4], early diagnosis and treatment are essential.

OA pathogenesis includes aging, obesity, joint trauma, joint instability, and heredity [5], which are related to intense mechanical stress on articular cartilage and lead to catabolic conditions within the joint [6,7]. Although many reports have suggested that repeated excessive mechanical strain may alter chondrocyte gene expression and induce the degradation of extracellular matrix through the upregulation of matrix metalloproteinases (MMPs) and a disintegrin and metalloproteinase with thrombospondin motifs (ADAMTS)-4/5, the precise mechanisms of mechanical stress-induced cartilage matrix degradation are still not fully understood [6,8].

The canonical Wnt/β-catenin pathway, which signals via low-density lipoprotein receptor-related proteins (LRPs) 5/6 and Frizzled, plays major roles in chondrocyte differentiation, and has been reported to be activated in OA cartilage [9,10,11], while inhibition of Wnt/β-catenin signaling is reported to reduce disease severity in a murine model of OA [12]. Other reports have shown that LRP4 antagonizes β-catenin signaling by competing with LRP5/6 in bone [13,14] and that late OA cartilage tissues express lower levels of LRP4 than normal or early OA tissues [15]. These results cumulatively suggest that LRP4 may have an important function in regulating LRP5/6 and their downstream β-catenin signaling in articular cartilage.

Agrin has long been reported to be a heparan sulfate proteoglycan which binds LRP4 and participates in neuromuscular synapse formation through the induction of acetylcholine receptor aggregation at postsynaptic sites [16,17]. Recently, the expression of Agrin in osteoblasts and chondrocytes and its involvement in differentiation have been reported; however, its function in articular cartilage remains unknown [18,19,20].

This study aimed to elucidate the function of LRP4 and Agrin in the process of human articular cartilage degeneration by subjecting human chondrocytes to mechanical stress and focusing on β-catenin signaling.

## 2. Results

### 2.1. Expression of LRP4 Increased in the Early Stages of OA and Decreased with Cartilage Degeneration, Whereas Agrin Was Consistently Increased with Cartilage Degeneration in Human Articular Cartilage Tissues

**Figure 1:**
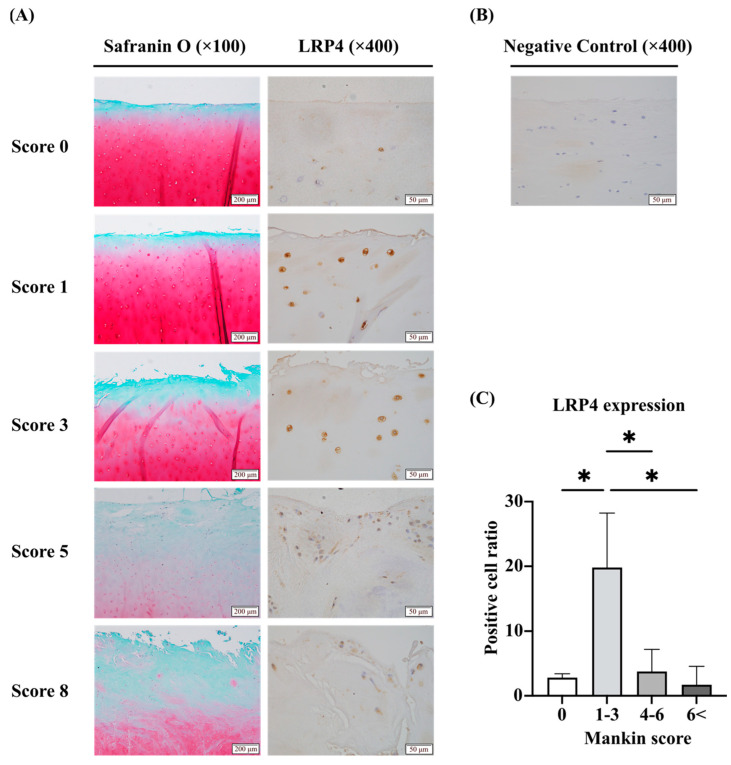
Immunohistochemical evaluation of low-density lipoprotein receptor-related protein (LRP) 4 expression in articular cartilage. (**A**) In the group with a Mankin score of 0, LRP4 expression was not clear. In samples with scores 1 and 3, expression was strongly positive. In samples with scores 5 and 8, expression was decreased. (**B**) No signal was observed in the negative control. (**C**) Tissues were divided into four groups according to their Mankin scores. The group with scores of 1–3 showed a significant large increase in the LRP4-positive cell ratio. * *p* < 0.0001. Scale bars are indicated in the figures.

**Figure 2:**
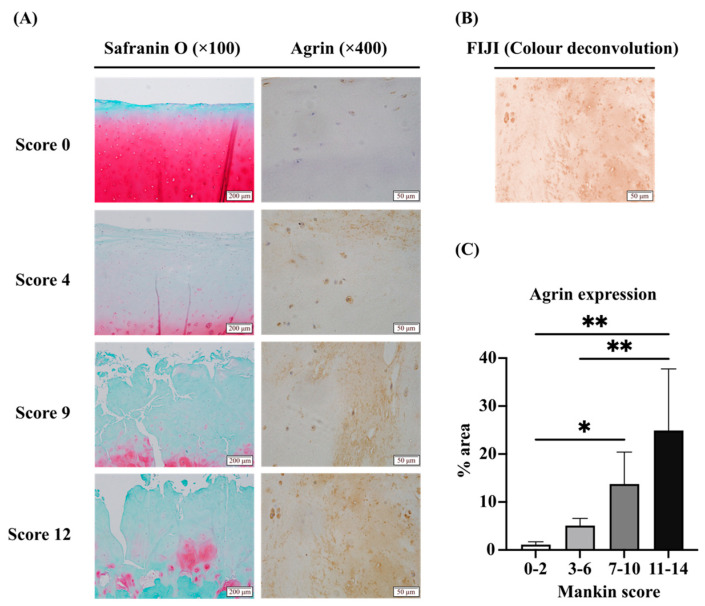
Immunohistochemical evaluation of Agrin expression in articular cartilage. (**A**) In samples with a Mankin score of 0, Agrin expression in the matrix was not clear. In samples with a score of 4, expression was weakly positive. In samples with a score of 9, expression was moderate. In samples with a score of 12, expression was high. (**B**) Images obtained from FIJI (ImageJ2 software) using the Colour deconvolution plugin. Diaminobenzidine (DAB) luminance was extracted and hematoxylin luminance was removed. (**C**) Tissues were divided into four groups according to their Mankin score degeneration classification (low: 0–2, mild: 3–6, moderate: 7–10, severe: 11–14). Agrin expression ratio (% area) increased with articular degeneration. * *p* < 0.01, ** *p* < 0.0001. Scale bars are indicated in the figures.

Immunohistochemical staining showed that LRP4 is expressed in human articular cartilage tissues and appears to respond to cartilage degeneration in the superficial layers of cartilage (Figure 1A,B). We divided the tissue samples into four groups according to the Mankin score as described below and evaluated them by calculating the LRP4 strong positive cell ratio (Figure 1C). LRP4 expression was increased in the early stages of OA and then decreased as cartilage degeneration progressed. Immunohistochemical staining of Agrin was conducted and focused on the cartilage matrix (Figure 2A). The samples were divided into four groups according to the Manikin score as described below and analyzed according to the percentage area of diaminobenzidine (DAB) staining using FIJI (ImageJ2 software, version 2.14.0/1.54f) (Figure 2B,C). Agrin expression was significantly increased as cartilage degeneration progressed. These results suggest that LRP4 and Agrin both respond to mechanical stress or inflammatory conditions in articular cartilage.

### 2.2. Expression of LRP4 and Agrin Were Regulated by Mechanical Stress with Cyclic Tensile Strain (CTS) in Human Articular Chondrocytes

**Figure 3:**
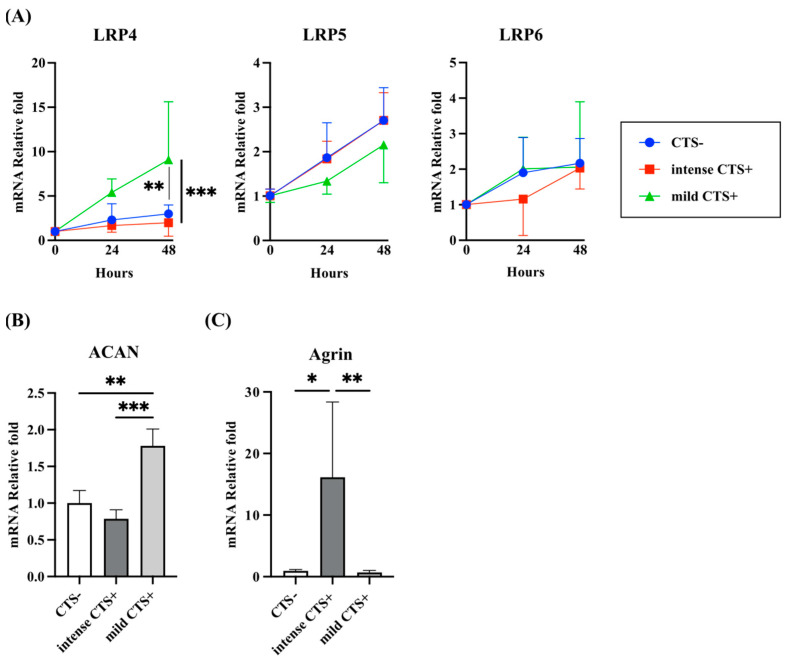
Relative gene expression in human articular chondrocytes after cyclic tensile strain (CTS). (**A**) After mild CTS, LRP4 expression was upregulated significantly compared to the CTS− and intense CTS+ groups. LRP5/6 expression was not affected by CTS intensity. (**B**) Aggrecan (ACAN) expression was upregulated at 48 h after mild CTS and downregulated after intense CTS. (**C**) Agrin expression was significantly upregulated at 48 h after intense CTS. * *p* < 0.05, ** *p* < 0.01, *** *p* < 0.001.

In normal human articular chondrocytes (NHACs), the mRNA expression of LRP4, LRP5, and LRP6 after application of mechanical stress by CTS was examined (Figure 3A). We set up intense CTS and mild CTS by adjusting the stretching stimulus as described below. LRP4 gene expression was upregulated after mild CTS and at 48 h after finishing CTS, a significant difference was observed between the control group and the intense CTS group. LRP5/6 expression did not differ with CTS intensity. Aggrecan (ACAN) expression, which is well known to be upregulated under anabolic conditions for chondrocytes, was upregulated after 48 h of mild CTS and downregulated after intense CTS (Figure 3B). Agrin expression was significantly upregulated after intense CTS (Figure 3C). From these results and the immunohistochemical results, LRP4 and Agrin expression seemed to be correlated with cartilage degeneration.

### 2.3. Agrin Treatment Upregulated the Gene Expression of LRP5/6 and Catabolic Factors in Human Articular Chondrocytes

**Figure 4:**
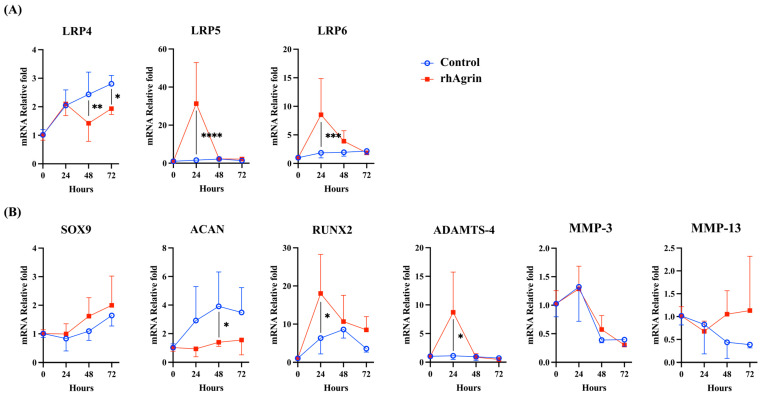
Effect of recombinant human (rh) Agrin treatment on gene expression in human articular chondrocytes. (**A**) LRP4 expression was downregulated at both 48 h and 72 h after rhAgrin treatment. LRP5/6 expression was upregulated at 24 h after rhAgrin treatment. (**B**) SRY-box transcription factor 9 (SOX9) expression seemed to be upregulated gradually, but did not show significant differences from the control group. ACAN expression was downregulated. Expression of Runt-related transcription factor 2 (RUNX2) and a disintegrin and metalloproteinase with thrombospondin motifs-4 (ADAMTS-4) was upregulated, whereas matrix metalloproteinases (MMPs) did not show significant differences. * *p* < 0.05, ** *p* < 0.01, *** *p* < 0.001, **** *p* < 0.001.

To elucidate the function of Agrin, we examined the effects of recombinant human (rh) Agrin treatment on NHAC. The mRNA expression of LRP4 and ACAN were downregulated, whereas LRP5/6, Runt-related transcription factor 2 (RUNX2), and ADAMTS-4 were upregulated. Expression of SRY-box transcription factor 9 (SOX9) seemed to be upregulated gradually, but did not show significant differences from the control group. MMP-3 and MMP-13 did not show significant differences (Figure 4A,B).

### 2.4. Agrin Promoted Nuclear Translocation of β-Catenin and SOX9 in Human Articular Chondrocytes

**Figure 5:**
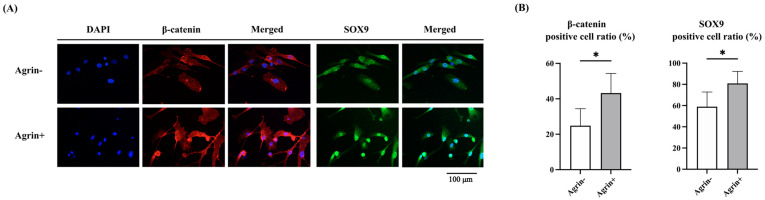
Immunocytochemical evaluation of the effect of rhAgrin treatment on human articular chondrocytes. (**A**) Double immunocytochemical staining of β-catenin and SOX9 nuclear translocation. (**B**) Translocation to nuclei of both β-catenin and SOX9 was increased after rhAgrin treatment. * *p* < 0.001. Scale bar = 100 μm.

**Figure 6:**
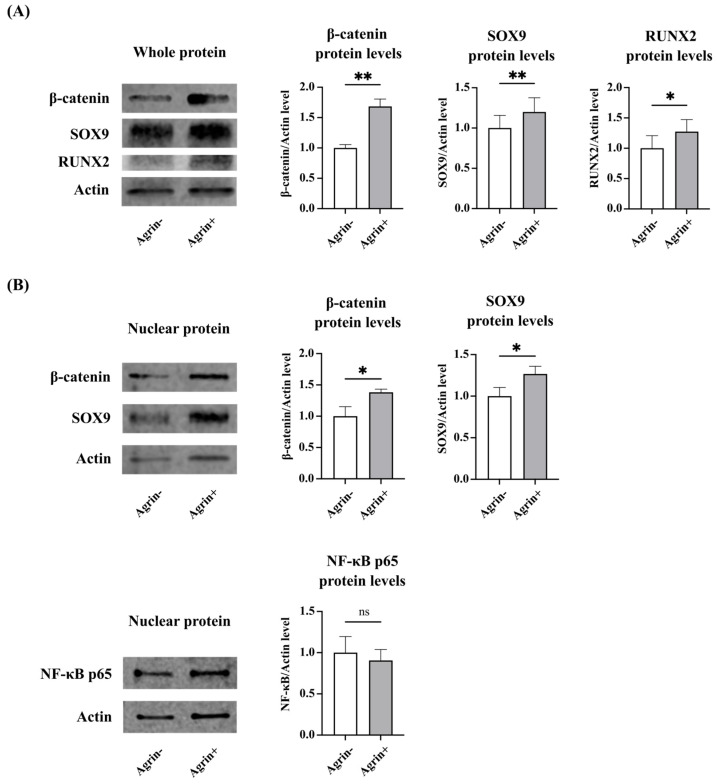
Western blot analysis of the effect of rhAgrin treatment on human articular chondrocytes. (**A**) At the whole protein level, the expression of β-catenin, RUNX2, and SOX9 were increased after rhAgrin treatment. (**B**) Analysis of nuclear extraction protein also showed that the expression of both β-catenin and SOX9 were increased after rhAgrin treatment. In contrast, analysis of nuclear extraction protein showed no significant difference in the expression of NF-κB p65. * *p* < 0.05, ** *p* < 0.01, ns: not significant.

Our results suggested that Agrin suppressed LRP4 function and promoted β-catenin signaling via LRP5/6. Next, we performed immunocytochemical evaluation and Western blot analysis for the localization of β-catenin at 24 h after rhAgrin treatment. According to the results of double immunocytochemical staining of NHAC, rhAgrin promoted the nuclear translocation of both β-catenin and SOX9 (Figure 5A,B). Western blot analysis revealed that both β-catenin and SOX9 expression were increased in whole protein and nuclear protein extracts after rhAgrin treatment. RUNX2 expression induced by β-catenin also increased at the protein level (Figure 6A,B). Since ADAMTS-4 gene expression was previously found to be upregulated after rhAgrin treatment, the nuclear translocation of NF-κB p65 was also examined through nuclear protein extraction, but there was no significant difference in expression.

### 2.5. Effects of Agrin Knockdown on Modulation of Intense CTS-Induced Genes in Human Articular Chondrocytes

**Figure 7:**
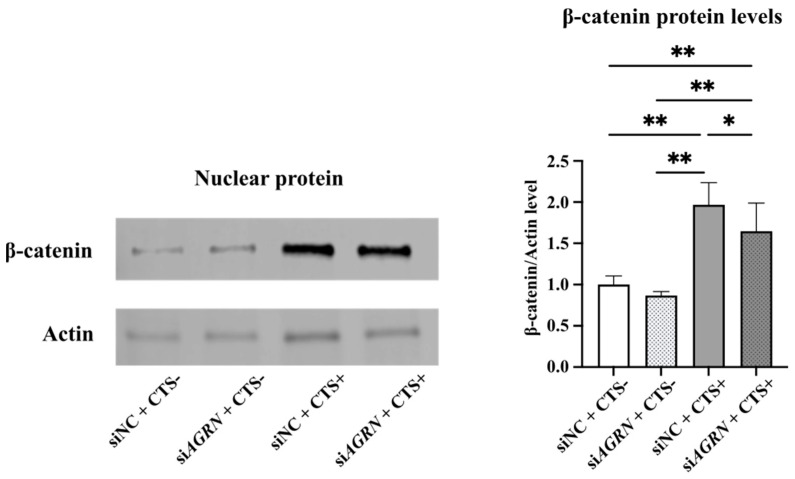
Combined effects of Agrin knockdown and intense CTS on β-catenin expression analyzed by Western blot. Expression of β-catenin was increased 24 h after application of intense CTS, but the si*AGRN* transfection + intense CTS group showed a trend towards reduced nuclear expression of β-catenin. * *p* < 0.001, ** *p* < 0.0001.

As upregulated Agrin with intense mechanical stress suppresses the function of LRP4 and promotes β-catenin nuclear translocation, inhibition of LRP4–Agrin binding may reduce β-catenin nuclear translocation in chondrocytes under intense CTS. To test this hypothesis, we combined intense CTS with Agrin knockdown and performed Western blot analysis in NHAC. Intense CTS significantly increased β-catenin nuclear translocation but Agrin knockdown by si*AGRN* transfection tended to partially reduce β-catenin nuclear translocation (Figure 7).

### 2.6. LRP4 Knockdown Showed a Similar Trend to Agrin After Treatment in Human Articular Chondrocytes

**Figure 8:**
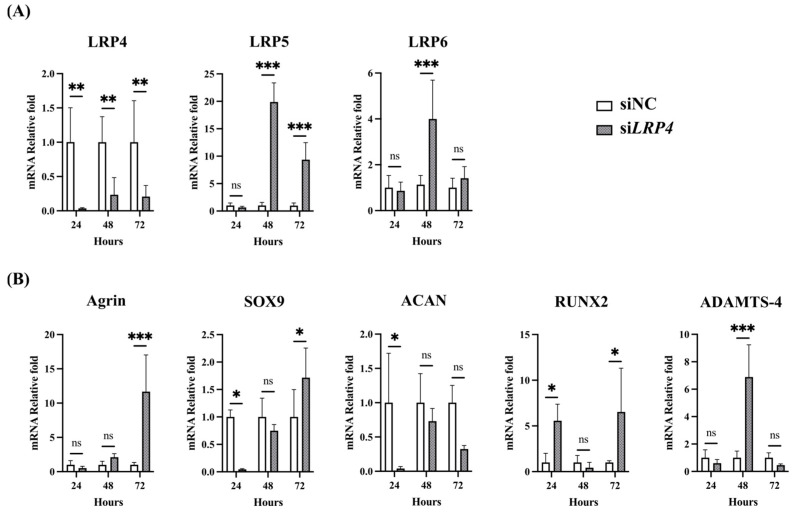
Effect of LRP4 knockdown on gene expression in human articular chondrocytes. (**A**) LRP4 expression was suppressed effectively at 24 h, 48 h, and 72 h after si*LRP4* transfection. LRP5 expression was significantly upregulated at both 48 h and 72 h. LRP6 expression was significantly upregulated at 48 h. (**B**) Agrin expression was significantly upregulated at 72 h. Expression of SOX9 and ACAN were significantly downregulated at 24 h, and then SOX9 expression was significantly upregulated, whereas ACAN was not upregulated at 72 h. Expression of RUNX2 was significantly upregulated at 24 h and then ADAMTS-4 was upregulated at 48 h. * *p* < 0.05, ** *p* < 0.01, *** *p* < 0.0001, ns: not significant.

**Figure 9:**
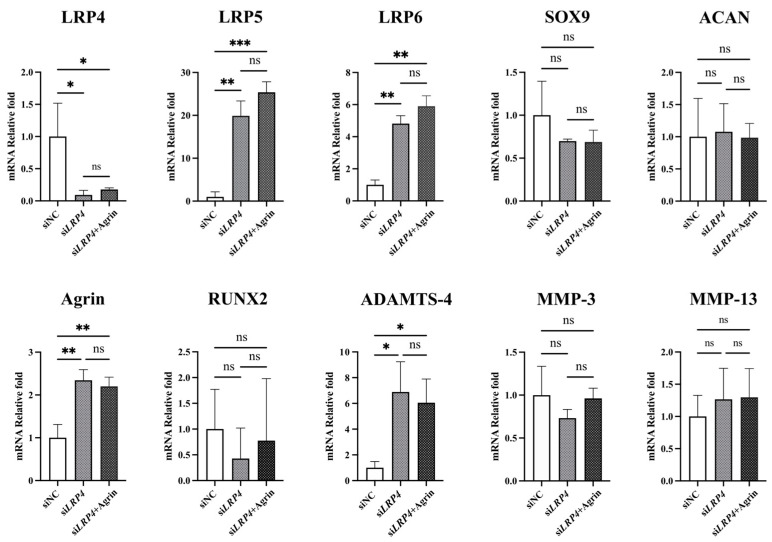
The combined effect of LRP4 knockdown and rhAgrin treatment on gene expression in human articular chondrocytes. On the expression of LRP4/5/6, Agrin, SOX9, ACAN, RUNX2, ADAMTS-4, MMP-3, and MMP-13, there were no significant differences between the si*LRP4* group and the si*LRP4* + Agrin group. * *p* < 0.05, ** *p* < 0.01, *** *p* < 0.001, ns: not significant.

To investigate LRP4 function, we performed LRP4 knockdown by si*LRP4* transfection. Interestingly, a similar trend was observed as with rhAgrin treatment, with the expression of LRP5/6, Agrin, RUNX2, and ADAMTS-4 upregulated, and ACAN levels not upregulated. SOX9 was not upregulated at 24 h but then was upregulated at 72 h (Figure 8A,B). From these results and the rhAgrin treatment results, we considered that Agrin might inhibit LRP4 function after binding as a ligand for LRP4. To examine whether Agrin has functions other than as an LRP4 ligand, we combined LRP4 knockdown with rhAgrin treatment. Cultured chondrocytes were treated with rhAgrin at 24 h after si*LRP4* transfection and collected 24 h later for analysis of gene expression (Figure 9). There were no significant differences in gene expression between the si*LRP4* group and the si*LRP4* + Agrin treatment group. These results suggested that Agrin has no function other than as an LRP4 ligand with chondrocytes under current experimental conditions.

## 3. Discussion

**Figure 10:**
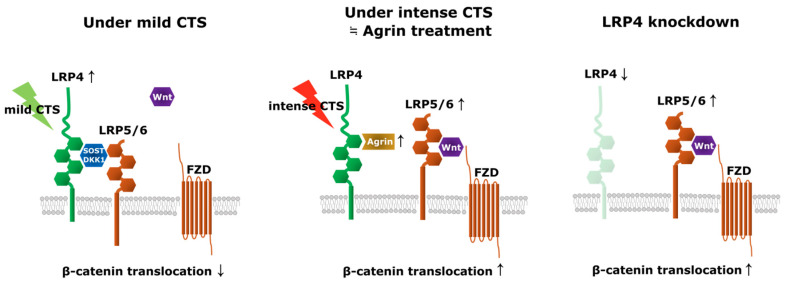
Modulation of LRP4 and Agrin under the current experimental conditions focusing on β-catenin signaling in human articular chondrocytes. Mild mechanical stress upregulated (↑) LRP4 expression in the superficial layer. Intense mechanical stress increased (↑) Agrin expression, leading to situations similar to LRP4 downregulation or LRP4 knockdown (↓), and then upregulated (↑) β-catenin translocation via LRP5/6. The LRP4 knockdown experiments showed that LRP4 negatively regulated LRP5/6 expression. FZD—Frizzled; SOST—Sclerostin; DKK1—Dickkopf-1.

This is the first study to report the upregulation of LRP4 expression by mild CTS in human chondrocytes. A previous study that examined early and late OA tissues showed lower LRP4 expression in late OA cartilage tissues [15], and another that examined LRP receptors showed that they are modulated by mechanical stress in rat chondrosarcoma cell pellets [21]. Asai et al. found that LRP4 induced gene expressions of extracellular matrix proteins of type II collagen (Col2a1), ACAN, and type X collagen (Col10a1), as well as the production of total proteoglycans in ATDC5 cells, whereas LRP4 knockdown had the opposite effects. In addition, LRP4 overexpression decreased β-catenin signaling in mouse ATDC5 chondrocytes, suggesting that LRP4 is an important regulator of extracellular matrix production and chondrocyte differentiation, acting by suppressing Wnt/β-catenin signaling [22]. Lietman et al. reported that the inhibition of Wnt/β-catenin signaling increased the expression of Col2a1 and proteoglycan 4 in chondrocytes isolated from patients with OA and ameliorated OA severity associated with reduced cartilage degeneration in vivo [12]. Geng et al. reported that LRP4 is a modulator of LRP6 in early forebrain [23], and other authors have found that LRP4 suppresses the function of LRP5/6 in bone [24,25,26]. The mechanism of LRP5/6 inhibition includes the possibility of LRP4 directly binding and internalizing LRP5/6 via sclerostin and the possibility of competing with Frizzled via sclerostin, dickkopf-1 (DKK1), and Wise [14,25,26,27]. Our experiments employing LRP4 knockdown in human articular chondrocytes also showed upregulation of LRP5/6, RUNX2, and ADAMTS-4 expression. Although it is difficult to discern whether LRP4 reacts directly due to mechanical stress or indirectly due to conditions triggered by mechanical stress, these reports and our findings suggested that the exertion of mild mechanical stress on cartilage causes upregulation of LRP4 expression, downregulation of LRP5/6 expression, and reduction in β-catenin signaling, leading to the upregulation of anabolic factors in chondrocytes (Figure 10).

The role of β-catenin signaling in cartilage seems to be diverse and involves interactions with various Wnt proteins [11]. β-catenin signaling is elevated in OA chondrocytes [9,10,11] and upregulates RUNX2 gene expression [28,29], which induces hypertrophic differentiation of chondrocytes and OA development [30,31], whereas adequate β-catenin signaling is reported to be important for cartilage maintenance, including chondrocyte proliferation and proteoglycan expression [11,32,33]. Furthermore, some reports found that β-catenin signaling upregulates not only RUNX2 but also SOX9, which is known to be a master regulator of chondrogenesis [29,34,35], and SOX9 transcriptionally activates the expression of inhibitors of β-catenin activity [36]. Because of the regulatory loop, RUNX2 and SOX9 may be sequentially upregulated under the current condition of the experiment. Our results of LRP4 knockdown and Western blot analysis of whole protein extracts following Agrin treatment also demonstrated increased expression of both RUNX2 and SOX9 proteins, which is presumed to be due to the activation of β-catenin signaling, and the results of immunocytochemistry and Western blot analysis of nuclear protein extracts following Agrin treatment showed increased nuclear translocation of both β-catenin and SOX9. These modulatory effects on genes and proteins by the regulatory loop may be essential for cartilage homeostasis.

Agrin, like sclerostin, DKK1, and Wise, is a ligand of LRP4 [13,37] and a heparan sulfate proteoglycan detected in osteoblasts and chondrocytes, which is related to their normal growth and differentiation [18,19,20]. Eldridge et al. reported increased Agrin expression in injured cartilage, and an Agrin-containing collagen gel induced the regeneration of bone and cartilage in sheep [38]. However, it was unknown whether Agrin responded directly to mechanical stress or to catabolic conditions caused by intense mechanical stress. In the current study, Agrin expression was upregulated along with cartilage degeneration in human OA cartilage tissues and with intense CTS in NHAC. Our results regarding Agrin treatment of chondrocytes showed upregulation of β-catenin and SOX9 nuclear translocation, but Eldridge et al. proposed the theory that Agrin–LRP4 binding downregulates β-catenin with the CREB-dependent signaling pathway in synovial joints [38]. The binding of Agrin to α-dystroglycan, which is important in discussing broader signaling effects in chondrocytes, has been reported to increase SOX9 expression and inhibit LRP5/6 function as a paracrine signal [20]. The results of this study are consistent with these findings. In addition, most of the studies on the interaction between Agrin and integrins are related to acetylcholine clustering at the neuromuscular junction, but the possibility that integrins mediate the interaction between Agrin and α-dystroglycan has been reported [39]. Thus, we examined whether Agrin has roles other than as an LRP4 ligand in chondrocytes by combining LRP4 knockdown with rhAgrin treatment. The results confirmed that Agrin has no function other than as an LRP4 ligand under the current experimental conditions. Since a similar trend was observed between the results of rhAgrin treatment and those of LRP4 knockdown, this suggests that Agrin–LRP4 binding might lead to situations similar to LRP4 downregulation or LRP4 knockdown in chondrocytes (Figure 10). In addition to the Wnt/β-catenin pathway and the CREB pathway, the NF-κB pathway might also be involved in the upregulation of catabolic factors such as ADAMTS and MMPs [28,29,40]; however, our results showed that rhAgrin treatment did not promote the nuclear translocation of NF-κB p65. Because it is possible that the inhibition of LRP4–Agrin binding might reduce intense CTS-induced β-catenin signaling, we examined the effect of combined intense CTS with Agrin knockdown. Intense CTS significantly increased β-catenin protein in nuclear extracts but si*AGRN* transfection only reduced β-catenin by a certain amount. According to these results, Agrin is expressed as a result of intense mechanical stress in articular cartilage and binds to LRP4 to induce catabolic factor expression via β-catenin signaling. In contrast, mild mechanical stress may induce the expression of anabolic factors, mediated by upregulated LRP4, suggesting that the inhibition of LRP4–Agrin binding in articular cartilage may represent a novel treatment for early OA.

There were several limitations to this study. First, chondrocytes were cultured in monolayers and evaluated by experiments in vitro. Second, we used a stretch machine to produce CTS, which is a simple model of OA chondrocyte behavior. Some reports suggest that cells respond differently to tensile and compressive stimuli. In vivo, chondrocytes are loaded not only by elongation but also compressive and shear stresses during joint motion, so there may be some difference in results compared to those in vivo. Third, we were able to collect only a limited number of clinical samples within the period, and finally, we were unable to perform in vivo experiments.

## 4. Materials and Methods

### 4.1. Cells and Cell Culture

Normal human articular chondrocytes from the knee joints (NHAC-kn) of a 19-year-old male and a 30-year-old male were purchased from Lonza (Walkersville, MD, USA) and human articular chondrocytes (HC-a) from a 23-year-old male and a 29-year-old male were purchased from StemBioSys (San Antonio, TX, USA). Cells were cultured at 37 °C in chondrocyte basal medium (Lonza, Walkersville, MD, USA) containing fetal b ovine serum (FBS), basic fibroblast growth factor (bFGF), R3 insulin-like growth factor (R3-IGF), transferrin, insulin, gentamicin, and amphotericin-B (CGM^TM^ BulletKit^TM^; Lonza, Walkersville, MD, USA). The attached cells were incubated at 37 °C in 5% CO_2_ in a humidified atmosphere and subcultured on type I collagen-coated polystyrene tissue culture dishes (Iwaki, Shizuoka, Japan). These cultured cells were used at passage 3 and cultured under starvation conditions of 2% FBS for 3 h before treatment and CTS.

### 4.2. Clinical Samples of Human Articular Cartilage Tissues

Adult human articular cartilage samples were obtained from six patients, of whom three underwent total knee arthroplasty for OA, one underwent total hip arthroplasty for OA, one underwent unicompartmental knee arthroplasty for OA, and one underwent above-knee amputation for trauma at our institution (Okayama University Hospital, Okayama, Japan). All procedures were approved by the Ethics Committee of Okayama University Graduate School of Medicine, Dentistry, and Pharmaceutical Sciences (No. 2312-033; approval date 1 December 2023). The patients comprised five women and one man aged 45–85 years at the time of the operation (Supplemental Table S1). These samples were fixed in 10% formalin solution for 24 h and then decalcified in 10% ethylenediaminetetraacetic acid (EDTA; pH 7.5) before embedding in paraffin blocks.

### 4.3. Histological Evaluation of Cartilage Destruction

Articular cartilage sections were stained with Safranin O-fast green and classified according to the histopathologic grading system described by Mankin et al. [41]. The Mankin score is a combined score assessing four parameters, the structure of articular cartilage (0–6), the cellularity of chondrocytes (0–3), proteoglycan staining (0–4), and the irregularity of tide marks (0–1). The histologic evaluation was performed by two independent blinded observers (SN and NS).

### 4.4. Immunohistochemical Evaluation of Articular Cartilage

Deparaffinized cartilage sections were pretreated with 10 mM citrate buffer (pH 6.0) in an autoclave at 90◦C for 5 min to retrieve the antigen. Endogenous peroxidase was blocked with 3% H_2_O_2_ in PBS at room temperature for 10 min. As the primary antibody, anti-LRP4 mouse monoclonal antibody (741704; Thermo Fisher Scientific, Waltham, MA, USA) diluted 1:100 and an anti-Agrin rabbit polyclonal antibody (ab85174; Abcam, Cambridge, UK) diluted 1:300 were used and incubated with the sections at 4 °C overnight. As secondary antibodies, Histofine Simple Stain MAX PO (M)/(R) (Nichirei Biosciences, Tokyo, Japan) was used at room temperature for 30 min. The reaction was visualized using DAB (Nichirei Biosciences) and counterstained with hematoxylin. Sections incubated with non-immune mouse serum were used as negative controls. The expression rate of LRP4 was evaluated in four groups of samples divided by the Mankin score (0, 1–3, 4–6, 6<), by calculating the percentage of strong positivity in superficial chondrocytes in 25 fields of view at ×400 magnification. Samples scoring 10 or above were excluded from this analysis as many sections lacked the superficial layer of cartilage. The expression rate of Agrin in the cartilage matrix was evaluated in the four groups divided by Mankin score (0–2, 3–6, 7–10, 11–14), by calculating the % area using FIJI (ImageJ2 software; NIH Image, Bethesda, MD, USA) in 30 fields of view at ×400 magnification. DAB luminance was extracted using the Colour deconvolution plugin.

### 4.5. CTS on Chondrocytes Cultured in Monolayer

Human articular chondrocytes were seeded into stretch chambers (STREX, Osaka, Japan) with a culture surface of 2 × 2 cm, coated with type I collagen at a density of 15,000 cells/cm^2^. After the cells were attached to the chamber surface, mechanical stress testing was performed using the ST-140-10 mechanical stretch system (STREX). The chambers were loaded into the incubator and subjected to uniform stretching stimuli [42]. Several reports have previously shown that hydrostatic pressure applied to articular cartilage during exercise changes the thickness of cartilage tissue and the associated rate of stretch of chondrocyte morphology [43,44]. Based on these reports and our previous studies [45,46], we set intense CTS (0.5 Hz, 10% elongation, 6 h) as the catabolic mechanical stress, and mild CTS (0.5 Hz, 5% elongation, 2 h) as the anabolic mechanical stress. Cells not subjected to mechanical stress were also seeded into stretch chambers and used as controls.

### 4.6. RT-qPCR Analysis

The cells were washed twice with PBS, and total RNA was purified using an RNeasy mini kit (Qiagen, Hilden, Germany). RNA samples (500 ng) were reverse-transcribed with Primescript RT master mix (Takara Bio Inc., Shiga, Japan), and the resulting cDNAs were used for real-time PCR using the QuantStudio™ 1 real-time PCR system (Thermo Fisher Scientific) with TaqMan Gene Expression Assays for human *LRP4* (Hs00391006_m1), *LRP5* (Hs01124561_m1), *LRP6* (Hs00233945_m1), *SOX9* (Hs00165814_m1), *ACAN* (Hs00165814_m1), *AGRN* (Hs00233992_m1), *RUNX2* (Hs00231692_m1), *ADAMTS4* (Hs00192708_m1), *MMP3* (Hs00968305_m1), *MMP13* (Hs00233992_m1), and *GAPDH* (Hs02786624_g1) (Thermo Fisher Scientific). Amplification of a housekeeping gene, GAPDH, was used to normalize the efficiency of cDNA synthesis and the amount of RNA. Each value obtained for the control cells (without CTS and treatment) was set to 1.

### 4.7. Treatment with Reagents

Recombinant human Agrin protein was purchased from R&D systems (6624-AG) and prepared at 50 ng/mL before use, following a previous report [20] and the experimental results for determining the concentrations of rhAgrin shown in the supplemental material (Supplemental Figure S1).

### 4.8. siRNA Transfection

siRNAs for *LRP4* and *AGRN* were purchased from Thermo Fisher Scientific (s8287, s8288, s8289, s534980, s534981, and s534982). The negative control siRNA was also purchased from Thermo Fisher Scientific (AM4611). Each nucleic acid and Lipofectamine RNAiMAX (13778075; Thermo Fisher Scientific) for transfection was diluted separately in reduced-serum medium Opti-MEM (31985062; Thermo Fisher Scientific) and incubated for 5 min, and then mixed and incubated for 10 min at room temperature to form the complex. Each complex was added to a medium containing cells and then seeded into culture dishes. The selection of siRNA for LRP4 and Agrin and the concentration of each nucleic acid are shown in the supplemental material (Supplemental Figures S2 and S3); 30 nM of s8289 as si*LRP4* and 10 nM of s534982 as si*AGRN* were selected.

### 4.9. Immunocytochemistry

Cells with or without treatment, according to the protocols described above, were fixed with 1% paraformaldehyde in PBS solution. The membranes of the culture chambers were then removed and incubated with anti-SOX9 antibodies (1:2000, ab185230, Abcam, Cambridge, UK) or anti-β-catenin antibodies (1:3200, #37447, Cell Signaling Technology, Danvers, MA, USA) for 2 h at room temperature. Bovine serum albumin-containing solutions without primary antibodies were used as negative controls. We used Alexa Fluor 488-conjugated antibody (goat anti-rabbit, ab150077, Abcam, Cambridge, UK) and Alexa Fluor 594-conjugated antibody (goat anti-mouse, ab150116, Abcam, Cambridge, UK) as secondary antibodies, and DAPI (ab104139, Abcam, Cambridge, UK) for nuclear staining. Cells were observed using a confocal laser scanning microscope (LSM780; Carl Zeiss, Jena, Germany) (Central Laboratory, Okayama University Medical School), and the protein expression level was evaluated by the proportion of nuclear-positive cells in 15 fields of view at ×100 magnification.

### 4.10. Western Blot Analysis

Cells were resuspended using an EPIXTRACT Nuclear Protein Isolation Kit II (Enzo Life Sciences, Farmingdale, NY, USA) to extract nuclear proteins. Each concentration of the protein extract was measured using a Bradford Protein Assay Kit (TaKaRa Bio Inc., Shiga, Japan) and was adjusted in each sample. Cell lysates were subjected to sodium dodecyl sulfate-polyacrylamide gel electrophoresis (SDS-PAGE) using a Mini-Protean^®^ Tris-glycine extended gel (Bio-Rad, Richmond, CA, USA) for approximately 1 h at 100 V before being transferred to PVDF membranes using Trans-Blot Turbo (Bio-Rad). The membranes were incubated with Odyssey^®^ Blocking Buffer (LI-COR Biosciences, Lincoln, NE, USA) for 1 h at room temperature and then incubated overnight at 4 °C with anti-SOX9 antibody (1:5000, ab185230, Abcam, Cambridge, UK), anti-β-catenin antibody (1:1000, #37447, Cell Signaling Technology), anti-RUNX2 antibody (1:1000, #12556, Cell Signaling Technology), or anti-NF-kB p65 (1:1000, #8242, Cell Signaling Technology) as the primary antibody. After washing with PBS + 0.1% Tween20, the membranes were incubated with IRDye goat anti-rabbit IgG (926-68071, LI-COR Biosciences, Lincoln, NE, USA) or IRDye goat anti-mouse IgG (926-32210, LI-COR Biosciences, Lincoln, NE, USA) as secondary antibodies at room temperature for 1 h. After washing with PBS + 0.1% Tween20, immunoreactive proteins were detected using the Odyssey Fc Imaging System (LI-COR Biosciences, Lincoln, NE, USA). Original, uncropped Western blot images are shown in Supplemental Figures (Supplemental Figure S4).

### 4.11. Statistical Analysis

All data are expressed as the mean ± 95% confidence intervals (CIs). Differences among individual sample groups were analyzed using a one-way analysis of variance (ANOVA) with Tukey’s multiple comparison tests. Differences between the two groups were compared by Student’s *t*-test. All differences were considered statistically significant at a *p* value < 0.05. All analyses were conducted using GraphPad Prism 10 (GraphPad Software, San Diego, CA, USA).

## 5. Conclusions

LRP4 expression was increased in the early stages of OA and then decreased with cartilage degeneration, while Agrin expression steadily increased. Mild CTS promoted LRP4 and ACAN expression, whereas intense CTS promoted Agrin expression in NHAC. Agrin treatment upregulated β-catenin, SOX9, and catabolic factors, and led to the suppression of LRP4 function. Our results also suggested that Agrin induced by intense mechanical stress interfered with the role of LRP4 in the inhibition of LRP5/6 and their downstream β-catenin signaling, leading to cartilage degeneration. These results support the idea that LRP4–Agrin binding might be a key modulator of chondrocyte homeostasis, and inhibition of Agrin binding to LRP4 may lead to a novel treatment for early OA.

## Data Availability

Data are contained within the article or supplementary material.

## Supplementary Materials

The following supporting information can be downloaded at: https://www.mdpi.com/article/10.3390/ijms26031007/s1.
