# Supplementary material for: LRP4 and Agrin Are Modulated by Cartilage Degeneration and Involved in β-Catenin Signaling in Human Articular Chondrocytes
Source: Int J Mol Sci. 2025 Jan 24;26(3):1007. doi: 10.3390/ijms26031007 (PMC11817251; doi:10.3390/ijms26031007)
Supplement: Supplementary file 1 [file ijms-26-01007-s001.zip › ijms-3257798-supplementary.pdf]

Individual patient data at the time of operation.

Supplemental Table S1. The patients comprised five women and one man aged 45–85 years at the time of the operation. TKA; total knee arthroplasty, THA; total hip arthroplasty, UKA; unicompartmental knee arthroplasty.

Patient No.	Age	Gender	injury/disease	procedure
1	69	F	injury	amputation
2	73	F	OA	TKA
3	85	F	OA	TKA
4	45	M	OA	THA
5	61	F	OA	TKA
6	52	F	OA	UKA

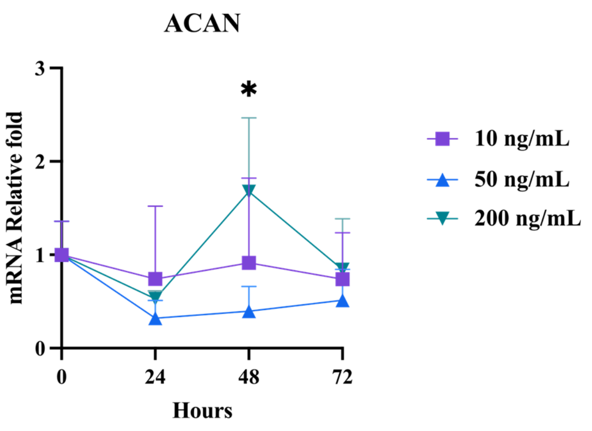

Supplemental Figure S1. RT-qPCR evaluation was performed to optimize rhAgrin reagent concentrations 24 h, 48 h and 72 h after treatment. Control cells at each time were set to 1. * $p < 0.01$ value of 50 ng/mL group relative to 200 ng/mL group.

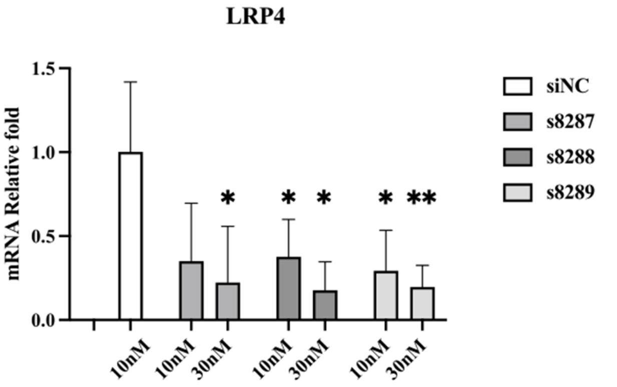

Supplemental Figure S2. RT-qPCR evaluation was performed for the selection of si*LRP4* reagent types and their concentrations 24 h after transfection. * $p < 0.05$ relative to siNC, ** $p < 0.01$ relative to siNC.

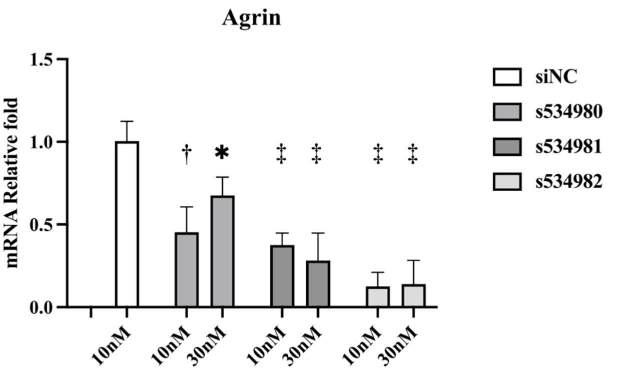

Supplemental Figure S3. RT-qPCR evaluation was performed for the selection of si*AGRN* reagent types and their concentrations 24 h after transfection. * $p < 0.01$ relative to siNC, † $p < 0.001$ relative to siNC, ‡ $p < 0.0001$ relative to siNC.

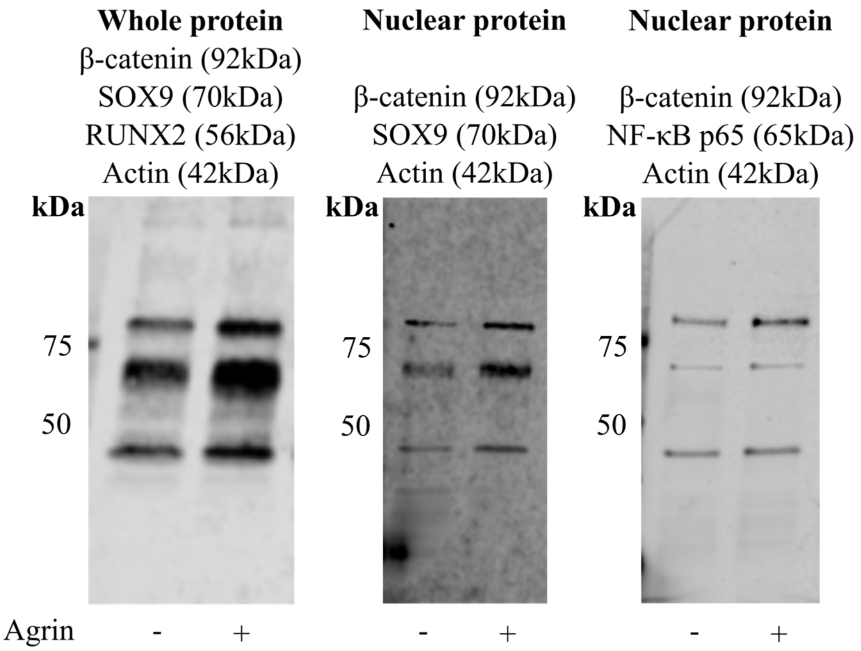

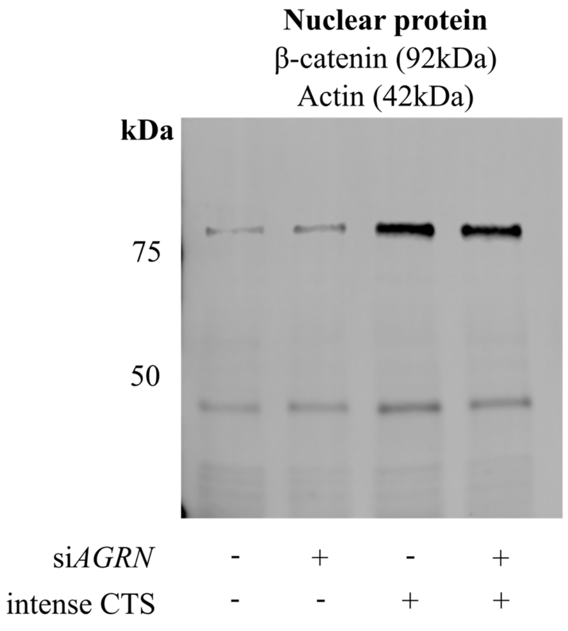

Supplemental Figure S4. Original, uncropped western blot images. The bands of β-catenin, SOX9, RUNX2, NF-κB p65 and Actin, and molecular weights of markers in normal human chondrocytes, are shown.
